# Human papilloma virus as causal in breast cancer

**DOI:** 10.3389/fonc.2026.1853427

**Published:** 2026-09-02

**Authors:** James S. Lawson, Wendy K. Glenn

**Affiliations:** School of Biotechnology and Biomolecular Sciences, University of New South Wales, Sydney, NSW, Australia

**Keywords:** breast cancer, causal, evidence, human papillomavirus, viruses

## Abstract

The aim of this review is to consider the role of high-risk human papillomaviruses (HPVs) in breast cancer. HPVs have established causal roles in cancers of the cervix, anus, penis, vagina, vulva, and oropharyngeal cancers. High-risk HPVs have been consistently detected in breast cancers in 23 countries. HPVs have been identified in 32.7% of breast cancers as compared to 7.6% of benign and normal breast controls. HPV-positive benign breast lesions can progress to HPV-positive malignancies, suggesting an early role of HPVs in carcinogenesis. HPV biological activity has been demonstrated by the detection of HPV transcripts, HPV oncogenic protein expression (HPV E6/E7), and HPV-induced koilocytes in breast cancer tissues. HPVs sourced from normal breast tissues and human milk can immortalise normal cultured breast epithelial cells. There is a plausible mechanism for the transfer of HPV material from the genital tract and cervix to the breast via blood and circulating exosomes. HPV-associated breast cancer is probably via a “hit and run” causal mechanism. The influence of HPV vaccination in reducing the incidence of breast cancer by 18% to 19% offers supportive evidence of causation. Other oncogenic viruses, including mouse mammary tumour virus, Epstein-Barr virus, and bovine leukaemia virus, may contribute to breast carcinogenesis independently or synergistically with high-risk HPVs. The evidence meets the extended Bradford Hill causal criteria. High-risk HPVs probably have a causal role in a subset of breast cancers.

## Introduction

Human papillomaviruses (HPVs) are established causes of cancers of the cervix, anus, penis, vagina, vulva, and oropharynx. Although HPV infection is common, progression to malignancy is relatively rare. Persistent infection of epithelial cells with high risk for cancer HPV types is the principal determinant of oncogenesis (1).

More than 200 HPV types have been identified, of which 15 are considered oncogenic (HPV 16, 18, 31, 33, 35, 39, 45, 51, 52, 56, 58, 59, 68, 72, and 81) (1). Among these, HPV16 and HPV18 account for the majority of HPV-related cancers. Transmission occurs primarily through sexual contact, but nonsexual routes—including blood, contaminated medical instruments (fomites), hand contact, and vertical transmission during childbirth—are also possible.

High-risk HPVs were first detected in breast cancers in 1992 by Di Lonardo and colleagues in Rome (2). Subsequent case–control studies have consistently confirmed that the prevalence of high-risk HPVs is significantly higher in breast cancers than in normal or benign breast tissue controls.

To evaluate causality, we applied an extended version of the classic A. Bradford Hill criteria (3). These include: (i) identification of the pathogen, (ii) epidemiological strength of association, (iii) consistency across studies, (iv) temporality, (v) exposure, (vi) experimental evidence, (vii) coherence, (viii) transmission, (ix) biological plausibility, and (x) oncogenic mechanisms.

We have assessed the evidence for a causal role of high-risk HPVs in breast cancer according to these criteria.

## Identification of high-risk for cancer HPVs in breast cancer

**Figure 1:**
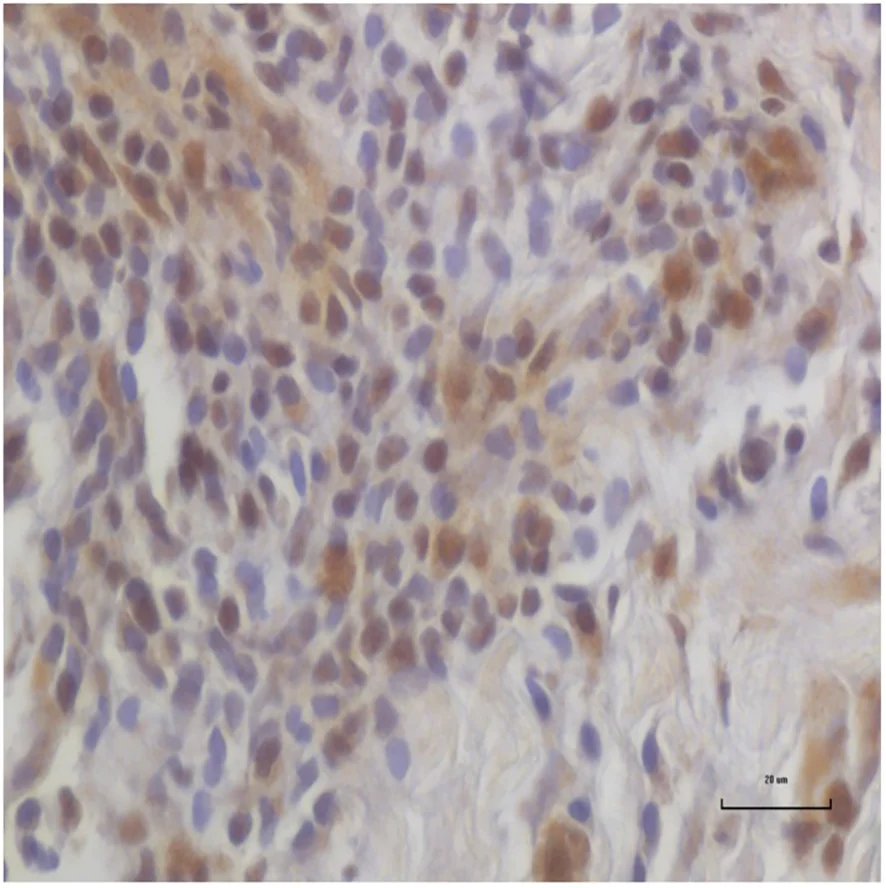
Oncogenic protein HPV E7 is shown (brown stains) in invasive breast cancer (5).

High-risk HPVs have been detected in breast cancers in multiple studies and countries (**Table 1**). Polymerase chain reaction (PCR) is the most commonly used method of detection. In one detailed investigation, HPV sequences were identified in 60% of breast cancers using both *in situ* and conventional PCR, complemented by next-generation sequencing (NGS) (4). In the same study, immunohistochemistry confirmed the presence of HPV oncoproteins. Oncoprotein HPV E7 in invasive breast cancer is shown in **Figure 1**.

**Table 1:** Summary of case–control studies reporting HPV prevalence in breast cancers in multiple countries.

Country	Study	Method	HPV breast cancer	HPV breast controls	Main HPV types	Significance
Australia	Heng 2009	PCR NP	8/26 31%	3/28 11%	16, 18	0.61ns
Australia	Glenn 2012	PCR IS	25/50 50%	8/40 20%	16,18	0.006s
Australia	Gannon 2015	PCR NP	13/80 17%	1/10 10%		0.002s
Australia	Lawson 2015	PCR NP PCR IS	27/40 66%	6/21 29%	16,18,52	0.001s
Brazil	Damin 2004	PCR PCR RT	25/101 25%	0/41 0%	16,18	0.001s
Brazil	Cavalcante 2018	PCR NP PCR MP	51/103 50%	15/95 16%	6,11,18, 31	0.001s
China	Yu 2000	PCR SBH	18/52 35%	0/15 0%	33,18	0.001s
China	He 2009	PCR (only HPV 16)	24/40 60%	1/20 5%	16	0.001s
China	Mou 2011	PCR NP, RDB	4/62 6%	0/46 0%	16,18	0.025s
China	Liang 2013	Hybrid capture	48/224 21%	6/37 16%	16,18,33,58	0.001s
China	Fu 2015	PCR TS	25/169 15%	1/83 1%	58	0.001s
China	Li 2015	PCR NP, PCR TS	3/187 2%	0/92 0%	6/16,18	0.157ns
China	Wang 2016	ISH TSP	52/146 36%	3/83 4%	16,18,58	0.001s
China	Zhang 2016	HPV GK	34/325 11%	4/100 4%	16,18	0.001s
Egypt	El-Sheik 2021	PCR RT	16/72 22%	0/15 0%	16,18	0.003s
Greece	Divani 2012	PCR NP, ISH, TSP	6/15 17%	0/35 0%	16,18	0.025s
India	Islam 2017	PCR	203/313 65%	2/2110%	16,18,33	0.001s
Iran	Sigaroodi 2012	PCR	15/58 26%	1/41 2%	16,18	0.002s
Iran	Ahanger 2014	PCR NP	22/65 34%	0/65 0%	16	0.001s
Iran	Manzouri 2014	PCR, HPVGK	10/55 18%	7/51 14%	16	0.083ns
Iran	Doosti 2016	PCR NP	10/87 23%	0/84 0%	16,18	0.001s
Iran	Malekpour 2018	PCR	8/98 8%	0/40 0%	16,18	0.008s
Iran	Khodabandehlou 2019	PCR L1, E7 genes	35/72 49%	5/31 11%	18	0.003s
Iran	Golrokh 2021	PCR NP	7/59 12%	0/15 0%	6,18	0.014s
Iran	Alinezhadi 2022	PCR NP	7/8 88%	2/9 22%	16	0.025s
Iran	Tavakolian 2023	PCR NP	9/40 23%	3/50 6%	18	0.014s
Iraq	Ali 2014	PCR IS, ISH	60/129 47%	3/41 7%	16,18,33	0.001s
Italy	Frega 2012	PCR	9/31 29%	0/12 0%	16,18	0.005s
Jordan	Al Hamad 2020	HPV RTK	21/100 21%	0/20 0%	16,18	0.007s
Jordan	Khasawneh 2024	PCR RTK	27/110 25%	0/30 0%	16,18	0.001s
Korea	Choi 2007	PCR	8/123 7%	0/31 0%	16,18,58	0.001s
Lebanon	Nagi 2021	PCR NP, HPV GK Oncogenic proteins	66/102 67%	5/14 36%	16,35,4,52,58	0.001s
Mexico	De Leon 2009	PCR	15/41 37%	0/43 0%	16,18	0.001s
Mexico	Mendizabal 2009	PCR	3/67 4%	0/40 0%	16,18,33	0.157ns
Morocco	El Amrani 2018	PCR TS, PCR MP	19/76 25%	1/12 8%	51,52,58	0.001s
Pakistan	Naushad 2017	PCR	45/250 18%	0/15 0%		0.001s
Spain	Delgado 2017	PCR, CLART, HPV DFC, RDB	130/251 52%	49/186 26%	16	0.001s
Taiwan	Tsai 2007	PCR, SBH	8/62 13%	2/32 6%		0.004s
Venezuela	Ladera 2017	HPV GTK	14/22 64%	1/22 4.5%	16,18,52,56	0.001
United Kingdom	Salman 2017	PCR, Western blot, dot blot	35/74 47%	11/36 18%	16,18, 35,45, 59	0.001s
Denmark	Bonlokke 2018	CISH	2/193 1.6%	1/100 1%	16, 56	0.157ns
United States	Guo 2021	PCR RT, HPV GK	25/56 45%	3/9 33%	11,18	0.005s
Pakistan	Awan 2025	PCR Oncogenic proteins	53/501 10.5%	2/110 2%	16, 18	0.001s
France	De Cremoux 2008	PCR	0/50 0%			
India	Hedau 2011	PCR	0/225 0%			
China	Zhou 2015	PCR	0/77 0%			
Tunisia	Hachana 2010	PCR TS, not nested,	0/123 0%			
Spain	Vernet-Tomas 2015	DNA immunoassay line probe assay	0/78 0%			
TOTALS			1192/3650 32.7%	146/1915 7.6%		0.001s

The references are listed in **Supplementary Table 1**.

ns, not significant; s, significant.

CISH, chromogenic *in-situ* hybridisation; CLART, amplification and microarray kit; HPV DFC, HPV direct flow chip; HPVGK, HPV Genotype Kit; HPVRTK, HPV real-time kit; ISH, *in-situ* hybridisation, PCR IS, PCR *in situ*; PCR, polymerase chain reaction; PCR MP, PCR multiplex; PCR NP, PCR nested primers - usually MY/GP to the L1 gene; RDB, reverse dot blot; PCR RT, PCR real time; SBH, southern blot hybridisation; TSP, type specific probes; PCR TS, PCR type specific.

The viral load of HPV in breast cancer tissue is extremely low, as demonstrated by Khan et al. (6). Using quantitative PCR, they estimated there are 5.4 HPV copies per 10,000 cells as compared with 130,000 per 100,000 cells in cervical cancers. This low viral load may be because of a “hit and run” causal mechanism whereby the virus is present in early but not late oncogenesis (7). This issue is considered below in the section on causal mechanisms.

There are well-recognised contamination problems associated with PCR technology. If there was contamination, it would be expected this would occur in both the cancer and controls, but this is not the case. In the studies listed in **Table 1**, the prevalence of HPVs in breast cancer is consistently higher than in controls.

### Next generation sequencing

In the U.S. Cancer Genome Atlas (TCGA) cohort, HPV gene sequences were detected in 50 of 855 breast cancer samples (8). Of these, 20 were high-risk HPV types (2.3%) and 30 were low-risk types (3.5%). The high-risk types identified included HPV16, HPV18, HPV31, HPV35, and HPV52. Furthermore, HPV proteins E1, E2, E4, E5, E6, E7, and L1 were detected in these HPV-positive breast cancer specimens. E1 and E2 are essential for HPV replication and play a significant role in the integration process and increase oncogenic E6 and E7 expression (9). These Next Generation Sequencing TCGA findings have been confirmed by Lawson et al. (5) The number of HPV transcripts in the TCGA cohort is very low in breast cancers (high-risk types 2.3% and low-risk types 3.5%) as compared to the identification of high-risk HPVs in breast cancers by PCR sequencing (most frequently 20%–30%) (5). The role of HPV E7 transcripts is considered in detail in the section below on oncogenic mechanisms.

## Epidemiology

### Case–control studies

In a meta-analysis, high-risk HPVs were detected in breast cancers in 43 of 48 case–control studies in 23 different countries (10). HPVs were not detected in five studies. These studies are included in **Table 1**. HPV16 and HPV18 were the most frequently identified types, reported in 87% of studies, while HPV33 was observed mainly among Asian populations. The prevalence of HPV-positive breast cancers was 32.7% compared to 7.6% in normal and benign breast tissue controls. The difference between breast cancer cases and controls is highly significant (*p* = 0.0001).

The study results from China and Iran are consistent. However, the results from other studies are inconsistent, varying from zero HPV identification in five studies to 88% (**Table 1**). Because the incidence of HPV cervical cancer is high in countries in which these five negative findings (France, India, China, Tunisia and Spain) occur, it is unlikely that these differences are due to low HPV infections. The difference in the incidence of HPV-positive breast cancer is probably due to variations in laboratory techniques. These differences include the use of fresh or fixed specimens, different PCR primers, timing of specimen collection and laboratory technical skills. The same problems exist with control specimens used in case control studies. In addition, control specimens may vary from normal breast tissues to benign breast tissues and non-malignant samples from tissues adjacent to breast cancers.

### Pooled case control data

HPVs were identified in 1,192 of 3,650 (32.7%) breast cancer cases. HPVs were identified in 146 of 1,915 (7.6%) normal and benign breast tissue controls. The differences are highly statistically significant (*p* = 0.001).

### Prospective cohort study

In a nationwide cohort of 61,872 women in Taiwan was followed for 7.2 years (**Table 1**). There were approximately 31,000 in the breast cancer group and 31,000 in the control group. Those women with HPV infection were 1.4 times more likely to develop breast cancer than women of the same age without HPV (11). Across all ages, HPV-positive women exhibited a 21%–36% higher risk of breast cancer compared with HPV-negative women (11).

The patients with HPV had an incidence rate of 12.5 per 10,000 person years, and those without HPV had an incidence rate of 9.81 per 10,000 person years. The relative risk of breast cancer in the HPV group compared with the non-HPV group was 1.27 (95% confidence interval 1.20–1.34). People older than 49 years were more likely to develop breast cancer. The risk of breast cancer in the 50−64 and ≥ 65 age groups was 1.67 times (95% CI = 1.56–1.80) and 1.36 times (95% CI = 1.32–1.52) higher, respectively, than that in patients younger than 49 years.

However, additional prospective cohort studies demonstrating that HPV infection precedes the development of breast cancer are required.

### HPVs in cervical and breast cancer

Women with HPV-associated cervical neoplasia are at increased risk of subsequent breast cancer compared with the general population (5, 12, 13). This is a consistent observation. The mean age at breast cancer diagnosis following cervical cancer is approximately 51 years—nearly a decade younger than the general average of 60 years (5). Although such cases represent a small proportion of breast cancers, they suggest a link between HPV infection in the cervix and the breast.

### HPV positive breast cancer is common in younger women

The average age of women with HPV-positive breast cancer is 38 years at the age of diagnosis as compared to 53 years for women with HPV-negative breast cancer (14). As HPV is a sexually transmitted virus and affects mostly young people, this association between positive HPV and younger age breast cancer is compatible with HPV having a role in breast cancer.

### HPVs in aggressive breast cancers

High-risk HPV DNA is more frequently detected in aggressive breast cancer subtypes, including triple-negative (44.4%) and HER2+ (48.4%) tumours, compared with Luminal A (23.9%) and Luminal B (30.1%) cancers (14).

### Multiple oncogenic viruses in breast cancer

HPVs are unlikely to act alone in causing breast cancer. Several oncogenic viruses may contribute to breast carcinogenesis, either independently or synergistically (15–19). In addition to HPVs, these include mouse mammary tumour virus (MMTV, the cause of breast cancer in mice), Epstein–Barr virus (EBV, associated with lymphomas and nasopharyngeal cancer), and bovine leukaemia virus (BLV, the cause of cancers in cattle (18). Each virus has been found more frequently in breast cancers than in benign or normal breast tissue controls. Case–control studies reported odds ratios of 13.40 for MMTV, 5.55 for HPV, 4.43 for EBV, and 2.57 for BLV, with MMTV showing the strongest association (18).

In a meta-analysis of 16 studies, Karen et al. showed a coinfection of HPVs and EBV in 14% of breast cancers (19). EBV and high-risk HPVs can be co-present in certain types of human malignancies, especially oral and breast cancer; consequently, oncoproteins of these viruses can cooperate to increase the invasive ability of such cancers via the initiation and amplification of the epithelial-mesenchymal transition (20, 21).

### Benign breast tissues can progress to breast cancer

**Figure 2:**
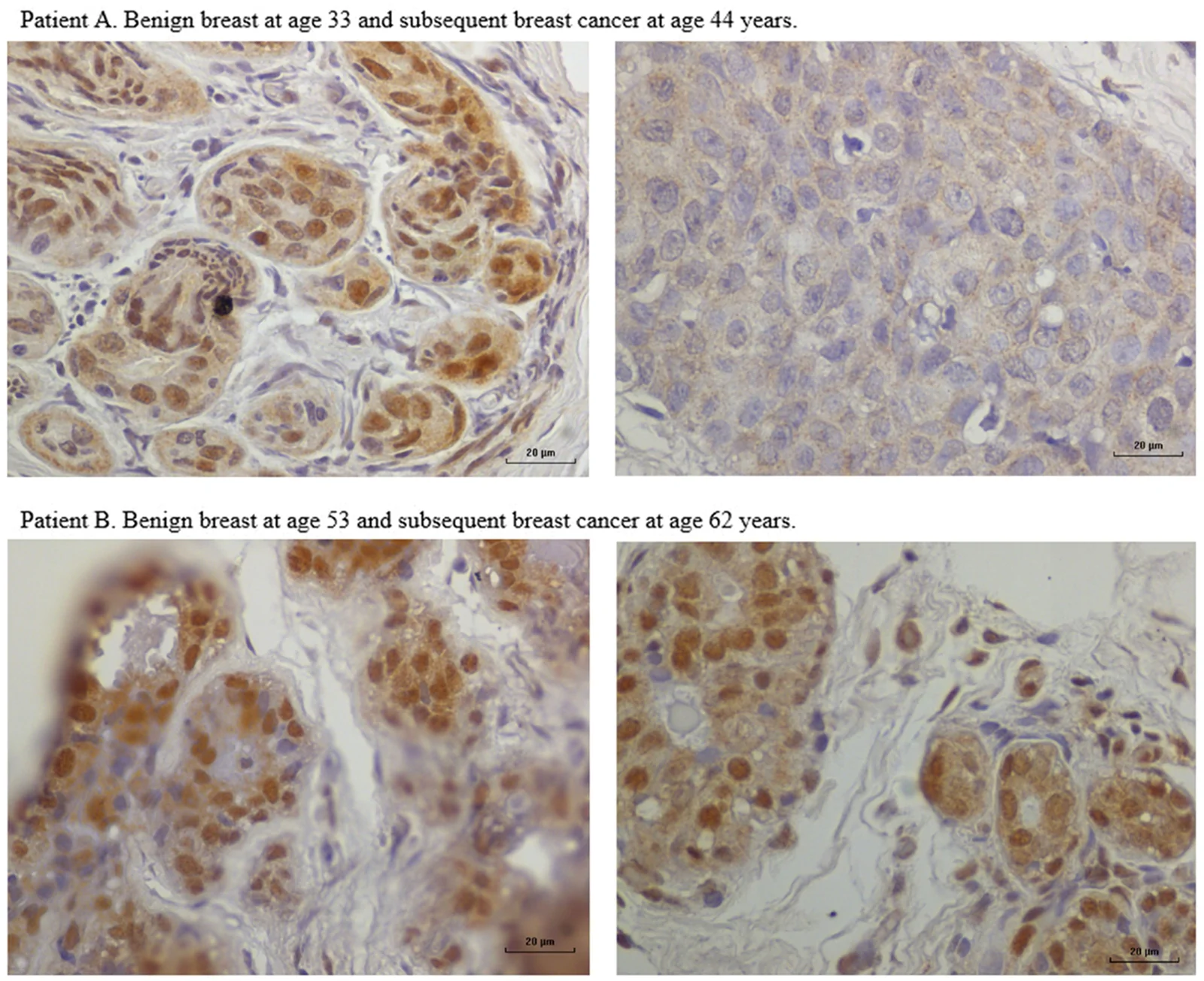
Immunohistochemical detection of HPV18 E7 protein in benign and subsequent breast cancer tissues. **(A)** Benign breast specimen at age 33 and subsequent invasive breast cancer at age 44. Strong nuclear E7 staining (brown) was observed in the benign specimen but absent in the subsequent cancer, suggesting an early “hit-and-run” role of HPV. **(B)** Benign breast specimen at age 53 and subsequent breast cancer at age 62. Strong nuclear E7 staining is present in both benign and malignant tissues.

High-risk HPV-positive benign breast conditions may progress to HPV-positive breast cancers (4, 20). Consistent HPV sequence patterns in both benign and subsequent malignant breast tissues from the same patients suggest that the same viral infection is present in both, supporting a potential role for HPV in malignant transformation (**Figure 2**). This is an important observation relative to causation.

### Mechanisms and early influences of high-risk HPVs in breast cancer

The mechanisms for HPV carcinogenesis in breast cancer probably differ from cervical cancer. HPVs may exert their oncogenic effects at an early stage of breast carcinogenesis, consistent with a “hit-and-run” mechanism. For example, HPV E7 oncoproteins have been detected in benign breast lesions but not in subsequent breast cancers in the same patients (4) (**Figure 2A**). However, these results are not uniform, as E7 expression has also been observed in both benign breast tissues and later breast cancer in some cases (4, **Figure 2B**).

There is experimental evidence in support of a “hit and run” HPV causal mechanism (7). Ferreira et al. used an HPV-driven oropharyngeal cancer model and CRISP to delete the HPV E7 gene. E7-deleted tumours initially regressed before the tumours recurred over time to develop new mutations (7).

In a study on experimental mice, Iwasaka et al. (1992) demonstrated that HPV viral sequences are not always required for the maintenance of a transformed state. They concluded HPV 18 can behave in a “hit and run” fashion (22).

Higher HPV genome numbers have been identified in premalignant as compared to malignant squamous cell carcinomas (23).

This “hit and run” mechanism appears to be different from HPV-associated cervical cancer. The presence of HPV E7 transcripts and HPV E7 oncoproteins has been shown experimentally to be a requirement for the maintenance of cervical cancer carcinogenesis (24).

Ngan et al. have demonstrated that HPVs may have an early oncogenic influence in breast cancer (20). In the Ngan et al. study, HPV E7 oncoproteins were identified in 72% of benign breast specimens but in only 30% of later breast cancer that developed in the same patients. This is shown in **Figure 2**.

A transcript is an RNA molecule that has been copied from a specific sequence of DNA and is required for the construction of oncogenic proteins. The number of HPV E7 oncoprotein transcripts in breast cancers is very low (high-risk types 2.3% and low-risk types 3.5%) as compared to the high number of HPV E7 transcripts in cervical cancers (8). This has been confirmed in a PCR-based study by Gannon et al. in which transcripts in HPV-associated breast cancer could not be identified (25).

The low number of HPV E7 transcripts contrasts with the identification of HPV E7 oncoprotein expression in 50% (12 of 24) of breast cancer specimens assessed by both immunohistochemistry and gene sequencing (4). HPV E7 oncoprotein expression identified by immunohistochemistry in invasive breast cancer is shown in **Figures 1**, **2**.

Accordingly, low HPV in breast cancer by the TCGA and other studies, absence of HPV transcripts in some breast cancers, HPV E7 in benign breast cancer followed by negative HPV E7 in later invasive breast cancers demonstrate it is probable that high risk HPVs can cause some breast cancers by a “hit and run” mechanism.

There is also evidence that HPV infections appear to enhance the biological activity of APOBEC3B genes, which are known to lead to an increased risk of breast cancer (26, 27).

The hypothesis that HPVs act early in breast oncogenesis may also explain why the prevalence of HPV-associated breast cancer is not increased in immunocompromised patients (28).

### HPV biological activity in breast cancer

HPV biological activity in breast cancer can be demonstrated in three principal ways: (i) detection of HPV transcripts, which indicate viral gene expression; (ii) expression of viral oncogenic proteins; and (iii) identification of HPV-associated koilocytes.

HPV RNA transcripts have been detected in breast cancers through the U.S. Cancer Genome Atlas (TCGA) program and confirmed by Lawson et al. (4, 8).

HPV oncogenic proteins have been confirmed in breast cancers using immunohistochemistry (**Figure 1**) (4).

**Figure 3:**
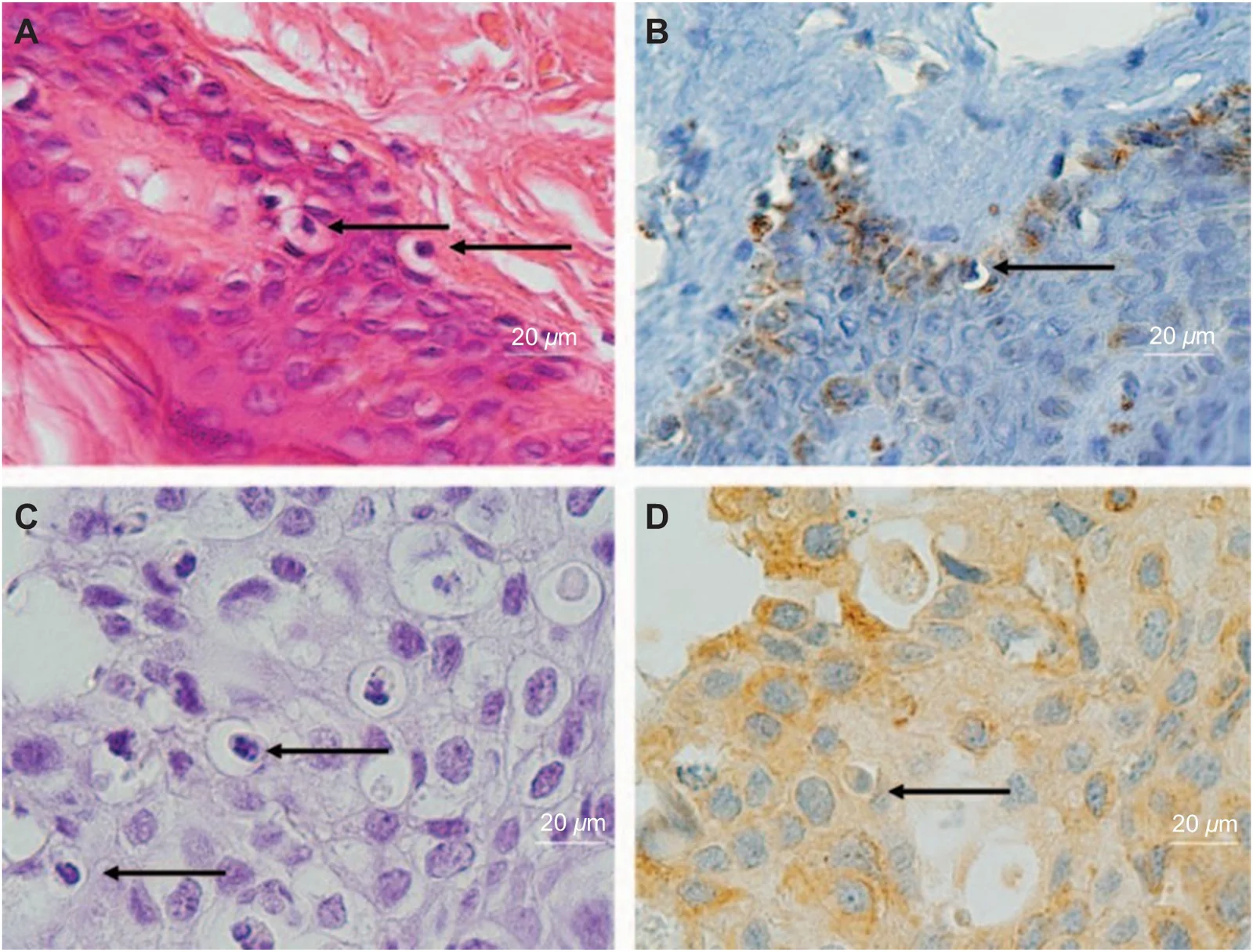
Koilocytes. Identification of HPV associated koilocytes in breast tissue. Arrows indicate representative koilocytes. **(A)** Haematoxylin and eosin (H&E) staining of normal breast skin. **(B)** Immunohistochemical detection of HPV E6 oncoprotein in the same normal specimen. **(C)** Haematoxylin staining (without eosin) of a ductal carcinoma insitu (DCIS) specimen. **(D)** Immunohistochemical detection of HPV E6 oncoprotein in the DCIS specimen.

In addition, HPV-induced koilocytes have been identified in breast cancer tissue (**Figure 3**) (29). In cervical cells, the early viral proteins E5 and E6 cooperate to produce koilocytosis (30). Because koilocytes form the cytological basis of the Papanicolaou (Pap) test, they are regarded as pathognomonic for HPV infection (30). Their presence in breast tissue provides strong evidence of HPV biological activity.

### Transfer of HPV from cervix to breast by exosomes (extracellular vesicles) and circulating blood

Exosomes, or extracellular vesicles, are membrane-bound particles secreted by most cell types, including cancer cells (31). When released from virus-infected cells, exosomes can carry viral proteins and nucleic acids. HPV E6 and E7 oncoproteins, as well as HPV DNA, have been identified in serum-derived exosomes (31–34). The protein composition of these exosomes closely mirrors that of their originating cancer cells.

High-risk HPVs and HPV oncoproteins have been identified in circulating blood mononuclear cells and blood plasma from patients with HPV-associated cervical cancer and blood from healthy blood donors (35–38).

These findings provide a plausible mechanism for the transfer of HPV material from the genital tract and cervix to the breast. However, there is no direct evidence that the presence of HPVs or HPV oncoproteins in extracellular vesicles or blood can lead to HPV-associated breast cancer.

### Breast epithelial cell immortalisation by HPVs

Cellular immortalisation represents an early indication of oncogenic transformation. Immortalised breast epithelial cells may serve as precursors for various forms of breast cancer (39–41).

In a pioneering study, Band et al. demonstrated that HPV16 and HPV18, isolated from breast reduction specimens, could immortalise normal human mammary epithelial cells (40). Whereas uninfected mammary epithelial cells typically undergo senescence after 18–20 passages, cells exposed to plasmids containing the complete viral genomes of HPV16 or HPV18 continued proliferating for more than 200 passages.

These findings were later confirmed in the same laboratory using HPV 16 E6 and E7 oncogenes, derived from normal breast tissue and human milk-derived, to immortalise normal mammary epithelial cells (41). Interestingly, different epithelial cell types required different viral oncogenes for immortalisation: HPV E6 alone, HPV E7 alone, or a combination of both (36). However, these mechanistic differences have not been directly correlated with distinct breast cancer subtypes.

When considered in isolation, the immortalisation studies by Band et al. and Wazer et al. do not prove HPV causation of breast cancer (40, 41). However, they add to the overall evidence.

### Influence of HPV vaccination on prevention of breast cancer

In a population-based study of young Brazilian women, HPV vaccination reduced the incidence of breast cancer by 18% to 19% (42). The purpose of this study was to determine the influence of HPV vaccination on cervical cancer in a low to moderate-income population. Breast cancer incidence was used as a potentially negative control. It was not anticipated that HPV vaccination would result in the reduction of breast cancer incidence.

The outcomes of this study have important limitations. It is based on population, not direct data. The women were young, aged 20 to 24 years. Breast cancer is very unusual in this age group. The women were all diagnosed within the Brazilian public health system (the sole provider of health services for approximately 70% to 80% of the population). On the other hand, because of the large population of 20- to 24-year-old women in Brazil (7.7 million), there were 1,475 breast cases identified in this age group in this study.

## Discussion and conclusions

The accumulated evidence can be evaluated using the extended A. Bradford Hill causal criteria.

### Identification of the pathogen

High-risk HPVs have been detected in breast cancers using diverse laboratory approaches, including standard and *in situ* PCR, next-generation sequencing, and immunohistochemistry for viral oncoproteins (4). This is a fundamental requirement for determining causation.

### Strength of the association, consistency, epidemiology

High-risk HPVs have been identified in breast cancers across 23 countries. Negative outcomes have been reported. The viral load of HPVs in breast cancer is very low, and their identification is technically difficult. This may account for these negative outcomes. In case-control studies shown in **Table 1**, the prevalence of HPV was 32.7% in breast cancers compared with 7.6% in normal and benign breast controls. This is a fourfold increase in cases as compared to controls and is highly statistically significant. HPV16 and HPV18 were the most common types. This is evidence for a role of high-risk HPVs in breast cancer.

Women with HPV infection have a 21%–36% higher risk of breast cancer. Women with HPV-positive cervical cancer are at increased risk of subsequent HPV-positive breast cancer. HPV prevalence is also elevated in aggressive subtypes such as triple-negative and HER2+ tumours. This is evidence that HPV infections at other locations can increase the risk of HPV-associated breast cancer.

### Pooled case control data

HPVs were identified in 1,192 of 3,650 (32.7%) breast cancer cases. HPVs were identified in 146 of 1,915 (7.6%) normal and benign breast tissue controls. The differences are highly statistically significant (*p* = 0.0001). In 38 (88%) of the 43 case-control studies the difference between HPV-positive breast cancer cases and controls was statistically significant.

A large prospective cohort study demonstrated that women with HPV were 1.4 times more likely to develop breast cancer than age-matched uninfected women (11). The identification of high-risk HPVs is significantly and consistently higher in breast cancers compared to normal and benign breast tissues (10). In China and Iran, with two exceptions high-risk HPVs are consistently higher in breast cancers compared to controls. The implication is that within populations of the same countries, where the cultures and sexual behaviours are similar, the pattern of high HPV in breast cancer and low HPV in controls is consistent. However, as shown in other countries, there is a wide variation in the prevalence of HPV in breast cancer. This is most likely because of the use of different laboratory techniques and collection and preservation of specimens.

### Multiple oncogenic viruses

Other oncogenic viruses, including mouse mammary tumour virus (MMTV), Epstein–Barr virus (EBV), and bovine leukaemia virus (BLV), may also contribute to breast carcinogenesis, either alone or in combination with HPV (18). Case–control studies indicate stronger associations for MMTV (odds ratio 13.40) compared with HPV (5.55), EBV (4.43), and BLV (2.57) (17).

### Temporality (timing)

HPV-positive benign breast lesions have been shown to progress to HPV-positive breast cancers (4). While this finding provides a confirmation of a temporal criterion, prospective cohort studies are required.

### Exposure and transmission

HPVs are primarily sexually transmitted. Viral material may be transferred from the cervix to the breast via exosomes and blood mononuclear cells containing HPV DNA and oncoproteins (32, 35). However, there is no direct evidence.

### Experimental evidence

High-risk HPVs isolated from breast tissue and human milk can immortalise normal mammary epithelial cells, providing evidence of oncogenic potential (40, 41). This evidence confirms the capacity of high-risk HPVs to potentially influence carcinogenesis in normal breast epithelial cells.

### Biological plausibility

Given that high-risk HPVs are established causes of cervical and other epithelial cancers, their involvement in breast cancer is biologically plausible. Supporting evidence includes detection of HPV transcripts, expression of HPV E6 and E7 oncoproteins, and the presence of HPV-induced koilocytes in breast tissue (4, 29).

### Oncogenic mechanisms

HPV oncogenic mechanisms may differ in breast as compared to cervical cancer. In cervical cancer viral integration into the host genome can drive overexpression of HPV E6 and E7, leading to p53 degradation and pRb inactivation. These mechanisms appear to operate similarly in some breast cancers. There is experimental evidence that “hit and run” phenomena may be involved in HPV-associated oesophageal cancers (7). The low viral load, the low number of HPV transcripts, and the presence of HPV oncoprotein in benign breast tissues but not in later breast cancer tissues in the same patients are suggestive of a “hit and run” phenomenon in up to 50% of HPV-associated breast cancers. There is also evidence that HPV infections appear to enhance the biological activity of APOBEC3B genes, which are known to lead to an increased risk of breast cancer (26, 27).

### Influence of HPV vaccination on prevention of breast cancer

In young Brazilian women HPV vaccination reduced the incidence of breast cancer by 18% to 19% (42). This is evidence that high-risk HPV infections can be associated with breast cancer. However confirmatory evidence is required.

### HPV. Causal or not causal in breast cancer

The most important evidence includes (i) identity of high-risk oncogenic HPVs in breast cancers, (ii) in 88% of 43 case-control studies, the difference between HPV-positive breast cancer cases and controls is statistically significant, (iii) in the only prospective cohort study, those women with HPV infections were 1.4 times more likely to develop breast cancer than women of the same age without HPV, and (iv) HPV vaccination reduces the incidence of breast cancer in young women.

When considered overall, the evidence meets the A. Bradford Hill criteria.

A. Bradford Hill concluded that “All scientific work is incomplete - whether it be observational or experimental. That does not confer upon us a freedom to ignore the knowledge we already have, or to postpone the action that it appears to demand at a given time.” (3) It follows that vaccination to prevent HPV infections should be encouraged with respect to breast and other cancers.

## Conclusion

The evidence supports high-risk HPVs as causal factors in a subset of breast cancers.
