# Supplementary material for: Human papilloma virus as causal in breast cancer
Source: Front Oncol. 2026 Sep 2;16:1853427. doi: 10.3389/fonc.2026.1853427 (PMC13577847; doi:10.3389/fonc.2026.1853427)
Supplement: Supplementary file 1 [file Table1.docx]

**Supplement references**

1. Heng B, Glenn WK, Ye Y, Tran B, Delprado W, Lutze-Mann L, et al. Human papilloma virus is associated with breast cancer. Br J Cancer. 2009;101:1345–1350.

2. Glenn WK, Heng B, Delprado W, Iacopetta B, Whitaker NJ, Lawson JS. Epstein-Barr virus, human papillomavirus and mouse mammary tumour virus as multiple viruses in breast cancer. PLoS One. 2012;7:e48788.

3. Gannon OM, Antonsson A, Milevskiy M, Brown MA, Saunders NA, Bennett IC. No association between HPV-positive breast cancer and expression of human papillomavirus transcripts. Sci Rep. 2015;5:18081.

4. Lawson JS, Glenn WK, Salyakina D, Delprado W, Clay R, Antonsson A, et al. Human papilloma viruses and breast cancer. Front Oncol. 2015;5:277. 19

5. Damin AP, Karam R, Zettler CG, Caleffi M, Alexandre CO. Evidence for an association of human papillomavirus and breast carcinomas. Breast Cancer Res Treat. 2004;84:131 137.

6. Cavalcante JR, Pinheiro LGP, Almeida PRC, Ferreira MVP, Cruz GA, Campelo TA, et al. Association of breast cancer with human papillomavirus infection in Northeast Brazil: molecular evidence. Clinics (Sao Paulo). 2018;73:e465.

7. Yu Y, Morimoto T, Sasa M, Okazaki K, Harada Y, Fujiwara T, et al. Human papillomavirus type 33 DNA in breast cancer in Chinese patients. Breast Cancer. 2000;7:33–36.

8. He Q, Zhang SQ, Chu YL, Jia XL, Wang XL. The correlations between HPV16 infection and expressions of c-erbB-2 and bcl-2 in breast carcinoma. Mol Biol Rep. 2009;36:807–9.

9. Mou X, Chen L, Liu F, Shen Y, Wang H, Li Y, et al. Low prevalence of human papillomavirus in Chinese patients with breast cancer. J Int Med Res. 2011;39:1636 -1644.

10. Liang W, Wang J, Wang C, Lv Y, Gao H, Zhang K, et al. Detection of high-risk human papillomaviruses in fresh breast cancer samples using the Hybrid Capture 2 assay. J Med Virol. 2013;85:2087–2092.

11. Fu L, Wang D, Shah W, Wang Y, Zhang G, He J. Association of human papillomavirus type 58 with breast cancer in Shaanxi Province of China. J Med Virol. 2015;87:1034 1040.

12. Li J, Ding J, Zhai K. Detection of human papillomavirus DNA in patients with breast tumor in China. PLoS One. 2015;10:e0136050.

13. Wang D, Fu L, Shah W, Zhang J, Yan Y, Ge X, et al. Presence of high-risk HPV DNA but indolent transcription of E6/E7 oncogenes in invasive ductal carcinoma of breast. Pathol Res Pract. 2016;212:1151–1156.

14. Zhang N, Ma ZP, Wang J, Bai HL, Li YX, Sun Q, et al. Human papillomavirus infection correlates with inflammatory Stat3 signaling activity and IL-17 expression in patients with breast cancer. Am J Transl Res. 2016;8:3214–3226.

15. El-Sheikh N, Mousa NO, Tawfeik AM, Saleh AM, Elshikh I, Deyab M, et al. Assessment of human papillomavirus infection and risk factors in Egyptian women with breast cancer. Breast Cancer (Auckl). 2021;15:1178223421996279.

16. Divani SN, Giovani AM. Detection of human papillomavirus DNA in fine needle aspirates of women with breast cancer. Arch Oncol. 2012;20:12–14.

17. Islam S, Dasgupta H, Roychowdhury A, Bhattacharya R, Mukherjee N, Roy A, et al. Study of association and molecular analysis of human papillomavirus in breast cancer of Indian patients: clinical and prognostic implication. PLoS One. 2017;12:e0172760.

18. Sigaroodi A, Nadji SA, Naghshvar F, Nategh R, Emami H, Velayati AA. Human papillomavirus is associated with breast cancer in the north part of Iran. Scientific World Journal. 2012;2012:837191.

19. Ahangar-Oskouee M, Shahmahmoodi S, Jalilvand S, Mahmoodi M, Ziaee AA, Esmaeili HA, et al. No detection of high-risk human papillomaviruses in a group of Iranian women with breast cancer. Asian Pac J Cancer Prev. 2014;15:4061–4065.

20. Manzouri L, Salehi R, Shariatpanahi S, Rezaie P. Prevalence of human papillomavirus among women with breast cancer in Isfahan, Iran. Adv Biomed Res. 2014;3:75.

21. Doosti M, Bakhshesh M, Zahir ST, Shayestehpour M, Karimi-Zarchi M. Lack of evidence for a relationship between high-risk human papillomaviruses and breast cancer in Iranian patients. Asian Pac J Cancer Prev. 2016;17:4357–4361.

22. Malekpour Afshar R, Balar N, Mollaei HR, Arabzadeh SA, Iranpour M. Low prevalence of human papillomavirus in patients with breast cancer, Kerman, Iran. Asian Pac J Cancer Prev. 2018;19:3039–3044.

23. Khodabandehlou N, Mostafaei S, Etemadi A, Ghasemi A, Payandeh M, Hadifar S, et al. Human papilloma virus and breast cancer: the role of inflammation and viral expressed proteins. BMC Cancer. 2019;19:61.

24. Golrokh Mofrad M, Sadigh ZA, Ainechi S, Faghihloo E. Detection of human papillomavirus genotypes, herpes simplex virus, varicella zoster virus and cytomegalovirus in breast cancer patients. Virol J. 2021;18-25.

25. Alinezhadi M, Makvandi M, Kaydani GA, Jazayeri SN, Charostad J, Talaeizadeh AT, et al. Detection of high-risk human papillomavirus DNA in invasive ductal carcinoma specimens. Asian Pac J Cancer Prev. 2022;23:3201–3207.

26. Tavakolian S, Faghihloo E. The prevalence of varicella zoster virus, herpes simplex virus type 2, and human papillomavirus in breast cancerous tissues and adjacent tissues in Iran. J Res Med Sci. 2023;28:65.

27. Ali SH, Al-Alwan NA, Al-Alwany SH. Detection and genotyping of human papillomavirus in breast cancer tissues from Iraqi patients. East Mediterr Health J. 2014;20:372–377.

28. Frega A, Lorenzon L, Bononi M, De Cesare A, Ciardi A, Lombardi D, et al. Evaluation of E6 and E7 mRNA expression in HPV DNA-positive breast cancer. Eur J Gynaecol Oncol. 2012;33:164–167.

29. Al Hamad M, Matalka I, Al Zoubi MS, Armogida I, Khasawneh R, Al-Husaini M, et al. Human mammary tumor virus, human papilloma virus, and Epstein-Barr virus infection are associated with sporadic breast cancer metastasis. Breast Cancer (Auckl). 2020;14:1178223420976388.

30. Khasawneh AI, Himsawi N, Sammour A, Al Shboul S, Alorjani M, Al-Momani H, et al. Association of human papilloma virus, cytomegalovirus, and Epstein-Barr virus with breast cancer in Jordanian women. Medicina (Kaunas). 2024;60:699.

31. Choi YL, Cho EY, Kim JH, Nam SJ, Oh YL, Song SY, et al. Detection of human papillomavirus DNA by DNA chip in breast carcinomas of Korean women. Tumour Biol. 2007;28:327–332.

32. Nagi K, Gupta I, Jurdi N, Jabeen A, Yasmeen A, Batist G, et al. High-risk human papillomaviruses and Epstein-Barr virus in breast cancer in Lebanese women and their association with tumor grade: a molecular and tissue microarray study. Cancer Cell Int. 2021;21:308.

33. de León DC, Montiel DP, Nemcova J, Mykyskova I, Turcios E, Villavicencio V, et al. Human papillomavirus in breast tumors: prevalence in a group of Mexican patients. BMC Cancer. 2009;9:26.

34. Mendizabal-Ruiz AP, Morales JA, Ramírez-Jirano LJ, Padilla-Rosas M, Morán Moguel MC, Montoya-Fuentes H. Low frequency of human papillomavirus DNA in breast cancer tissue. Breast Cancer Res Treat. 2009;114:189–194.

35. El Amrani A, Gheit T, Benhessou M, McKay-Chopin S, Attaleb M, Sahraoui S, et al. Prevalence of mucosal and cutaneous human papillomavirus in Moroccan breast cancer. Papillomavirus Res. 2018;5:150–155.

36. Naushad W, Surriya O, Sadia H. Prevalence of EBV, HPV and MMTV in Pakistani breast cancer patients: a possible etiological role of viruses in breast cancer. Infect Genet Evol. 2017;54:230–237.

37. Delgado-García S, Martínez-Escoriza JC, Alba A, Martín-Bayón TA, Ballester-Galiana H, Peiró G, et al. Presence of human papillomavirus DNA in breast cancer: a Spanish case control study. BMC Cancer. 2017;17:320.

38. Tsai JH, Hsu CS, Tsai CH, Su JM, Liu YT, Cheng MH, et al. Relationship between viral factors, axillary lymph node status and survival in breast cancer. J Cancer Res Clin Oncol. 2007;133:13–21.

39. Ladera M, Fernandes A, López M, Pesci-Feltri A, Ávila M, Correnti M. Presence of human papillomavirus and Epstein-Barr virus in breast cancer biopsies as potential risk factors. Gac Mex Oncol. 2017;16:103–108.

40. Salman NA, Davies G, Majidy F, Shakir F, Akinrinade H, Perumal D, et al. Association of high-risk human papillomavirus and breast cancer: a UK-based study. Sci Rep. 2017;7:43591.

41. Bønløkke S, Blaakær J, Steiniche T, Høgdall E, Jensen SG, Hammer A, et al. Evidence of no association between human papillomavirus and breast cancer. Front Oncol. 2018;8:209.

42. Guo H, Idrovo JP, Cao J, Roychoudhury S, Navale P, Auguste LJ, et al. Human papillomavirus detection by chromogenic in situ hybridization and p16 immunohistochemistry in breast intraductal papilloma and breast carcinoma. Clin Breast Cancer. 2021;21:e638–e646.

43. Awan UA, Khattak AA, Ahmed N, Guo X, Akhtar S, Kamran S, et al. An updated systematic review and meta-analysis on human papillomavirus in breast carcinogenesis. Front Oncol. 2023;13:1219161.

44. de Cremoux P, Thioux M, Lebigot I, Sigal-Zafrani B, Salmon R, Sastre-Garau X, et al. No evidence of human papillomavirus DNA sequences in invasive breast carcinoma. Breast Cancer Res Treat. 2008;109:55–58.

45. Hedau S, Kumar U, Hussain S, Shukla S, Pande S, Jain N, et al. Breast cancer and human papillomavirus infection: no evidence of HPV etiology of breast cancer in Indian women. BMC Cancer. 2011;11:27.

46. Zhou Y, Li J, Ji Y, Ren M, Pang B, Chu M, et al. Inconclusive role of human papillomavirus infection in breast cancer. Infect Agent Cancer. 2015;10:36.

47. Hachana M, Ziadi S, Amara K, Toumi I, Korbi S, Trimeche M. No evidence of human papillomavirus DNA in breast carcinoma in Tunisian patients. Breast. 2010 Dec;19(6):541-4.

48. Vernet-Tomás M, Mena M, Alemany L, Bravo I, De Sanjosé S, Nicolau P, et al. Human papillomavirus and breast cancer: no evidence of association in a Spanish set of cases. Anticancer Res. 2015;35:851–856. In r
